# Advanced 3D printed model of middle cerebral artery aneurysms for neurosurgery simulation

**DOI:** 10.1186/s41205-019-0048-9

**Published:** 2019-08-01

**Authors:** Ruth G. Nagassa, Paul G. McMenamin, Justin W. Adams, Michelle R. Quayle, Jeffrey V. Rosenfeld

**Affiliations:** 1 Department of Anatomy and Developmental Biology, Monash University, Clayton, VIC Australia; 2 Monash Institute of Medical Engineering, Monash University, Clayton, VIC Australia; 3 Department of Neurosurgery, The Alfred Hospital, Melbourne, VIC Australia; 4 Department of Surgery, F. Edward Hébert School of Medicine, Uniformed Services University, Bethesda, MD USA

**Keywords:** Neurosurgical training, Anatomical models, Aneurysm, 3D printing, Simulation

## Abstract

**Background:**

Neurosurgical residents are finding it more difficult to obtain experience as the primary operator in aneurysm surgery. The present study aimed to replicate patient-derived cranial anatomy, pathology and human tissue properties relevant to cerebral aneurysm intervention through 3D printing and 3D print-driven casting techniques. The final simulator was designed to provide accurate simulation of a human head with a middle cerebral artery (MCA) aneurysm.

**Methods:**

This study utilized living human and cadaver-derived medical imaging data including CT angiography and MRI scans. Computer-aided design (CAD) models and pre-existing computational 3D models were also incorporated in the development of the simulator. The design was based on including anatomical components vital to the surgery of MCA aneurysms while focusing on reproducibility, adaptability and functionality of the simulator. Various methods of 3D printing were utilized for the direct development of anatomical replicas and moulds for casting components that optimized the bio-mimicry and mechanical properties of human tissues. Synthetic materials including various types of silicone and ballistics gelatin were cast in these moulds. A novel technique utilizing water-soluble wax and silicone was used to establish hollow patient-derived cerebrovascular models.

**Results:**

A patient-derived 3D aneurysm model was constructed for a MCA aneurysm. Multiple cerebral aneurysm models, patient-derived and CAD, were replicated as hollow high-fidelity models. The final assembled simulator integrated six anatomical components relevant to the treatment of cerebral aneurysms of the Circle of Willis in the left cerebral hemisphere. These included models of the cerebral vasculature, cranial nerves, brain, meninges, skull and skin. The cerebral circulation was modeled through the patient-derived vasculature within the brain model. Linear and volumetric measurements of specific physical modular components were repeated, averaged and compared to the original 3D meshes generated from the medical imaging data. Calculation of the concordance correlation coefficient (ρ_c_: 90.2%–99.0%) and percentage difference (≤0.4%) confirmed the accuracy of the models.

**Conclusions:**

A multi-disciplinary approach involving 3D printing and casting techniques was used to successfully construct a multi-component cerebral aneurysm surgery simulator. Further study is planned to demonstrate the educational value of the proposed simulator for neurosurgery residents.

## Background

Neurosurgery trainees are finding it increasingly difficult to obtain operative experience as the primary operator in aneurysm surgery [1]. Good quality cadaver dissection opportunities are also not readily available for neurosurgery residents. Simulation is emerging as a useful training aid for neurosurgery [2]. The treatment of cerebral aneurysms requires specialized skill development and proficient use of micro-instruments. Furthermore, any advance in neurosurgical training methods is of potential value to both neurosurgeons and patients [3]. Operative caseload of neurosurgical trainees forms an extensive role in training, alongside surgical simulation in the development of primary neurosurgical techniques [4]. Current simulation methods include the use of human cadavers, large animal models, medical manikins and virtual simulation with haptic feedback [5, 6]. However, these models rarely simulate the entire procedure or provide realistic haptic feedback [7, 8]. A 3D model mimicking human tissue would allow trainees to go through the basic operative steps of specific procedures and navigate through anatomical landmarks enabling effective training with the supervision of superiors in a safe environment [9]. Simulation-based training has been shown to improve non-technical skills including cognitive and interpersonal skills that can be overlooked in surgical training [10, 11]. Such improvements in the context of neurosurgical trainees would decrease medical error and potentially improve patient outcomes [12]. 3D printed models have been demonstrated to accurately replicate patient-specific vascular structures [11]. Such models have been shown to be valuable in endovascular coiling simulation where anatomical complexities are detected through medical imaging and therefore require the determination of a preoperative tactile approach [13]. They can assist in improving the understanding of spatial anatomy configuration, particularly in cases of challenging vasculature [13].

In many cases, 3D printed simulation models have not replicated the cerebral aneurysm together with adjacent arteries [14], vessel lumen [15] and haptic properties. The ideal model for cranial surgery simulation should include: head anatomy with surface landmarks; ability to be positioned in a three point head clamp; realistic scalp, bone, dura and brain; and accurate representation of pathology [16].

Here we describe our development of a cerebral aneurysm simulator that includes the entire human head and relevant surgical anatomy for complete simulation of cerebrovascular surgery.

## Methods

To improve the reproducibility and cost efficacy, the left side of the brain was designed with the surgical approach and realistic pathology of a middle cerebral artery (MCA) aneurysm at the M1/M2 junction. Due to the inability to obtain all necessary components of a comprehensive simulator from a singular patient dataset, each component was developed from various patient medical imaging and computational 3D models that obtained optimal clarity of the desired anatomy.

**Fig. 1:**
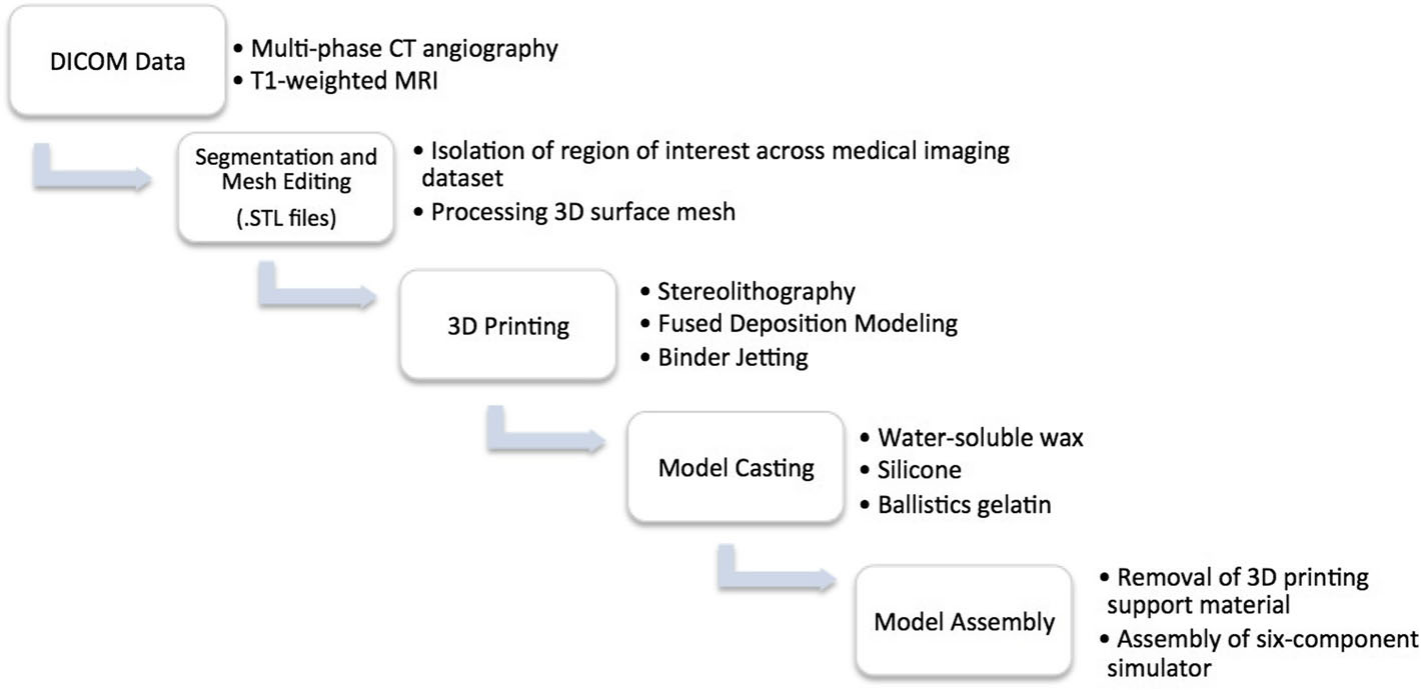
Flowchart presents the workflow for simulator development. Initially, DICOM data was imported into segmentation software in which the region of interest was isolated. The 3D rendered mesh was processed. The generated .STL files were assigned to a 3D printer to print anatomical models and moulds. The moulds were used to cast soft-tissue models in various materials. Post-print processing involved the removal of 3D printing support materials and assembly of the six-component simulator

Non-anatomical geometries were designed on the 3D computer graphics and computer-aided design application software Rhinoceros, Robert McNeel & Associates, Educational edition, version 4.0. These geometries were integrated in order to interlock multi-component models into a fixed position and form supporting structures for moulds. The method of manufacture is outlined in Fig.1. The parameters of each 3D printed model and properties of the 3D print-driven casts are listed in Tables 1 and 2 respectively.

**Table 1:** Parameters of each 3D printed model

	Vasculature Model	Cranial Nerve Model	Brain Mould (Left and Right)	Skull Model (Two-components)	Skin Mould
3D Printer	Formlabs Form 2	MakerBot Replicator 2X	3D Systems ZPrinter 650	3D Systems ZPrinter 650	3D Systems ZPrinter 650
Substrate	Photopolymer resin (V3 FLGPWH03)	Polymaker Polyflex™	Zp131 Powder, Zb61 Binder	Zp131 Powder, Zb61 Binder	Zp131 Powder, Zb61 Binder
Printer Resolution (mm)	0.05	0.15	0.10	0.10	0.10
Print Time (hours)	3.5	0.8	13.5	11.5	20.5

**Table 2:** Mechanical and physical properties of 3D print-driven casts

Mechanical and Physical Properties	Dragon Skin™ 10 Fast	Ecoflex™ 00–10	Clear Ballistics Medical Gel #4
Product Type	Silicone Rubber – Platinum Cure	Silicone Rubber – Platinum Cure	Synthetic Gelatin
Mix Ratio By Volume	1A: 1B	1A: 1B	–
Mix Ration By Weight	1A: 1B	1A: 1B	–
Pot Life (minutes)	8	30	–
Cure Time (hours)	1.25	4	3
Hardness	10 Shore A	00–10 Shore A	3.3 Shore OO
Density (kg/m^3^)	–	–	834
Melting Point (°C)	–	–	125
Tensile Strength (psi)	475	120	–
100% Modulus (psi)	22	8	–
Cost	US $16.11/ lb	US $16.11/ lb	US $31.81/ lb

### Developing vascular models with Lumina

This study made use of CT angiograms (multiphase CT2) of patients who presented to the Alfred Hospital (Melbourne, Victoria) with cerebral aneurysms in 2017. Data selection was restricted to cases of aneurysms that occurred at the MCA, with a defined aneurysm neck, aneurysm size greater than 5 mm and cases that were surgically clipped. A single dataset was chosen that met these requirements. The data belonged to a male patient who presented with a saccular aneurysm at the M1/M2 junction. CT angiography was performed on a Siemens Axiom Artis Angio System at a slice thickness of 0.36 mm (512 slices) at the Alfred Health Radiology Service (Melbourne, Victoria). For reconstructive purposes the patient aneurysm dataset was merged with a pre-existing 3D computational model derived from a patient CT angiogram obtained from the Centre of Human Anatomy Education (CHAE), Department of Anatomy and Developmental Biology (ADB), Monash University, which contained a clear visual of the internal carotid artery (ICA).

**Fig. 2:**
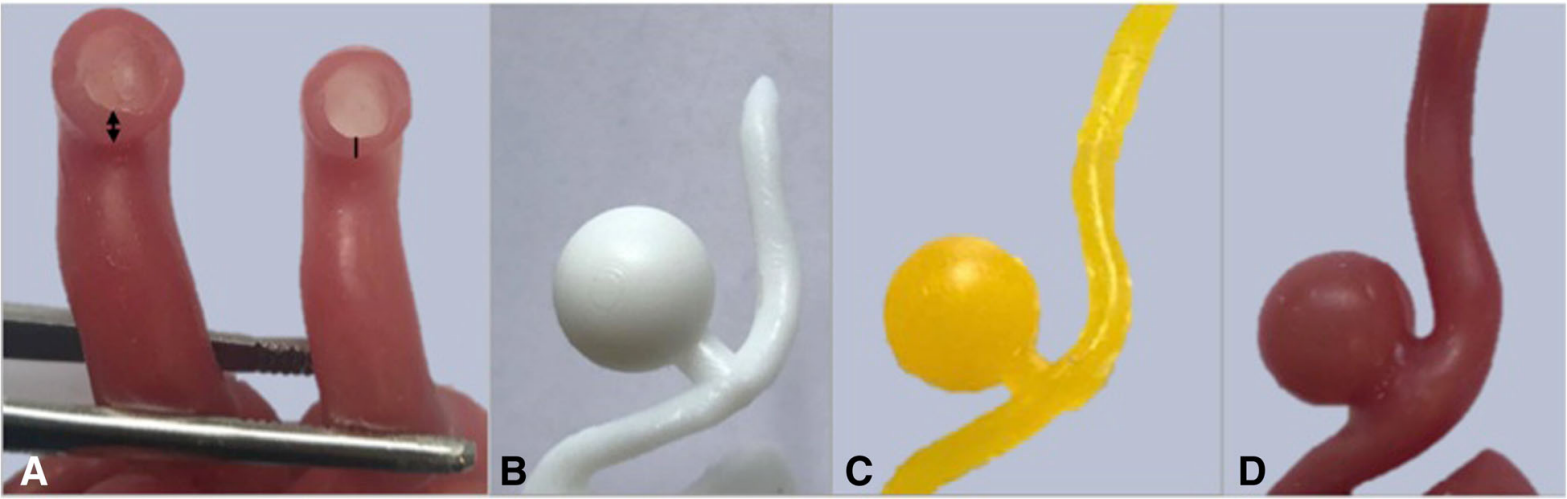
**a** Terminal branches of the patient-derived vasculature lumen (left MCA) in which two layers of silicone (line) produced a thinner, more accurate representation of vessel wall thickness than that of three layers (double sided arrow). **b** 3D print of CAD aneurysm model used to develop **c** a wax cast and **d** hollow silicone model

The medical image files (IMA files) were converted to Digital Imaging and Communications in Medicine (DICOM) data using the free access software 3D Slicer (version 4.6). The DICOM data was then imported into segmentation software Mimics, Materialise Software, version 19.0 Leuven, Belgium to isolate the cerebral vasculature across all slices of the scan. A semi-automatic thresholding process reported the Hounsfield units (HU) and tissue with a density ranging 2848 to 12807 HU were segmented. Following segmentation, the geometries were rendered as a 3D mesh and converted to a Binary, Little Endian standard tessellation language (.STL file). The files were edited on Geomagic Control, 3D Systems where the left ICA, MCA and anterior cerebral artery (ACA) were collectively partitioned from the Circle of Willis. The models were printed on the Formlabs Form 2 high definition desktop 3D printer in photopolymer resin (V3 FLGPWH03). A two-part mould of the model was created using Dalchem Mould Making Silicone Rubber SRT-30®. A negative of the model was achieved and the hollow cavity was filled with Sol-U-Carv Directions water-soluble wax. The wax model was then brush-coated with Smooth-On™ Dragon skin 10® platinum cure silicone rubber with Smooth-On™ pigment additive Silc Pig in the color Blood (PMS 7421C). Two or three layers of the silicone was applied to achieve a vessel wall (Fig. 2a). The model was placed in water allowing the wax to dissolve through evacuation points to establish a vessel lumen. Vessel wall thickness was measured using the Mitutoyo Sliding Caliper (Absolute, 500-171-30).

### Computer-aided design aneurysm

An aneurysm was constructed on Geomagic Control using the Feature tool to form a sphere 10 mm in diameter. To construct an aneurysmal neck a cylinder with a diameter of 5 mm was constructed in correspondence with the width of the parent vasculature. The CAD aneurysm was merged using the Combine tool at the MCA. Following the same method, the model was printed, moulded and cast. In order to replicate the sensitivity of an aneurysmal vascular wall, the aneurysm was coated in a single layer of silicone and two layers were coated on the remaining vasculature (Fig. 2b-d).

### Patient aneurysm

The aneurysm and parent vessel geometries were designed separately to facilitate the alteration of aneurysm models and allow easier post-printing manipulation. On Geomagic Control the aneurysm was partitioned with the Trim With Plane tool to segment the MCA from M1 to M2. The isolated patient aneurysm and secondary vasculature model were merged using the Combine tool to visualize the appropriate orientation to be achieved post-printing. Following the same method, the model was printed, moulded and cast in wax. Melted Sol-U-Carv wax facilitated the adhesion of the patient aneurysm to the vasculature model. One layer of silicone was coated on the aneurysm and two layers were coated on the remaining vasculature.

### Circulatory system

The Waterpik® Waterflosser Ultra (WP100) was used to replicate the cerebral circulation. The tip of the apparatus that projects fluid in a pulsatile manner was introduced to the vascular model and tubes used to extend the terminal branches to form an output. The tubing was connected to the water reservoir of the Waterpik to form a circuit. Smooth-On Ultimate Blood® Base was added to the water reservoir to mimic the color of blood. The circulatory system projected through the patient-derived vasculature within the brain model.

### Cranial nerve model

**Fig. 3:**
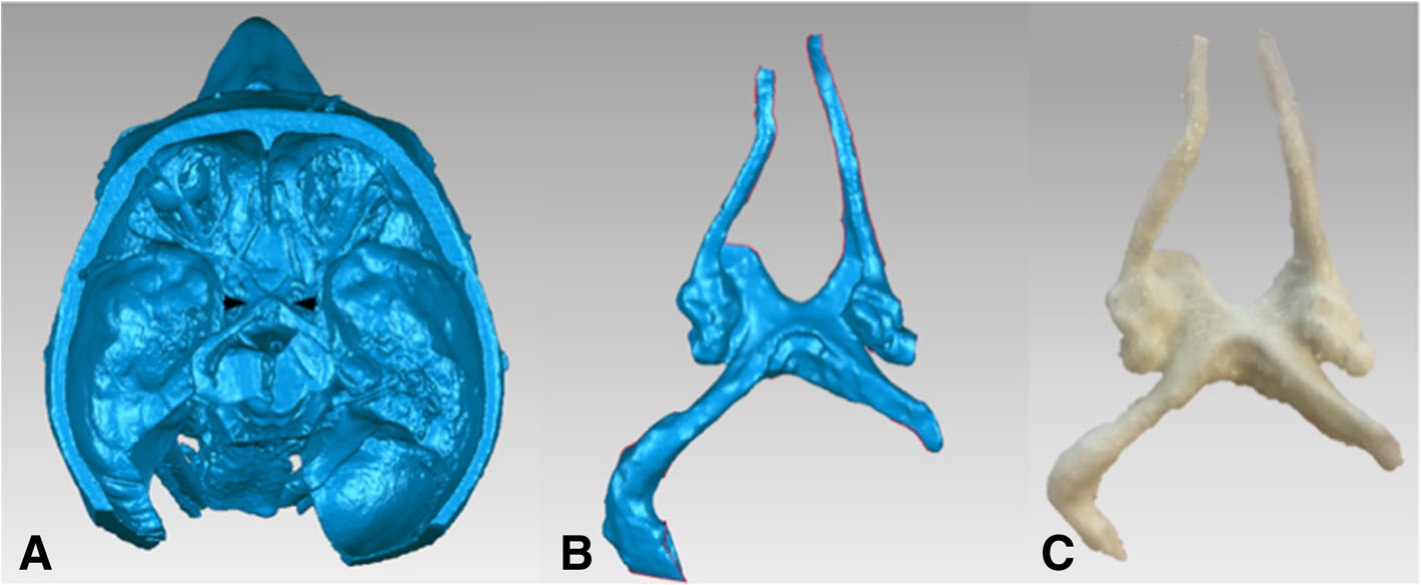
**a** Computational model of dissected specimen (MP1670) with the optic chiasm, optic nerve and olfactory tract (between arrows) that were isolated **b** as a separate mesh and **c** 3D printed through FDM technology to achieve a flexible 3D printed model

The optic chiasm, optic nerve and olfactory tract were included to serve as anatomical landmarks for intraoperative navigation. Pre-existing data of a dissected specimen (MP1670 Head and Visceral Column of the Neck) from the CHAE, Department of ADB, Monash University, was used to isolate this desired anatomy. The .STL file was imported into Geomagic Control where a box developed on Rhinoceros was used to eliminate structures of the cranial cavity using the Boolean (Subtract One) tool, until the optic chiasm, optic nerve and olfactory tract remained. The open surfaces were resolved with the Fill All (Flat) tool. The model was printed on the MakerBot Replicator 2X desktop 3D printer in thermoplastic elastomer Polymaker Polyflex™ (Fig. 3). The print was adhered to the cranial base of the skull model using super glue.

### Brain model

The brain model was developed from a patient T1-weighted MRI scan obtained from The Cancer Imaging Archive (TCIA) at www.cancerimagingarchive.net. The medical imaging data was chosen based on the number of slices (512 coronal; 512 sagittal; 212 transverse) and slice thickness (slice increment 1.0 mm; pixel size 0.51 mm) for optimal data extraction. Data selection was restricted to MRI scans that lacked superficial pathological abnormalities that could impact the overall interface of the brain.

The data was imported into Aviso Lite (FEI Software, version 9.0.1.) accessed through Multi-modal Australian Sciences Imaging and Visualisation Environment (MASSIVE). Segmentation of the brain was performed using Magic Wand to select data within the threshold range of 1245 to 1284 HU. Precise isolation of boundaries involved manually tracing the complete geometry of the brain using the Lasso tool across all planes. Following segmentation, the Generate Surface tool was used to construct a 3D surface model through Unconstrained Smoothing. The final surface model was imported into Geomagic Wrap® (2015.1.1).

Using Trim With Plane tool, the brain was trimmed along the longitudinal fissure to separate the two cerebral hemispheres. To achieve a dissectible Sylvian fissure that could be mechanically manipulated during simulation, the brain mesh was trimmed at tunnels formed at the anterior ramus and posterior ramus of the lateral sulcus. The dimensions of the Sylvian fissure were taken using the Analysis Measure Distance tool.

**Fig. 4:**
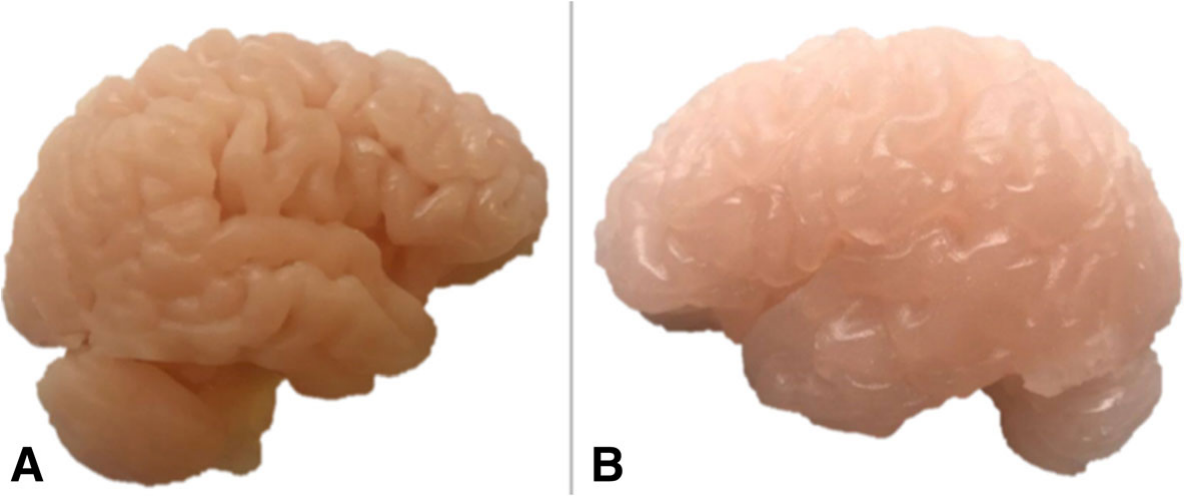
**a** Right cerebral hemisphere cast in Clear Ballistics gel 10% with firmer material properties to that of the **b** left cerebral hemisphere cast in Clear Ballistics medical gel #4

Each hemisphere was inverted using the Invert Planes tool in order to have the desired anatomy on the exterior surface. A box developed on Rhinoceros was merged with each hemisphere using the Combine tool to develop a block mould. The moulds were printed on the ZPrinter 650, 3D Systems powder-based printer. The mould of the left hemisphere was used to cast a replica in Clear Ballistics medical gel #4 with the lowest density to achieve haptic properties of brain tissue. The right was cast in Clear Ballistics gel 10% to provide a higher shore hardness surface to prevent slumping of the left hemisphere within the simulator. Both gels were combined with Silc Pig® pigment additives in the color Flesh Tone and White (PMS White) to replicate the color and consistency of brain tissue (Fig. 4).

### Meninges model

#### Dura mater

**Fig. 5:**
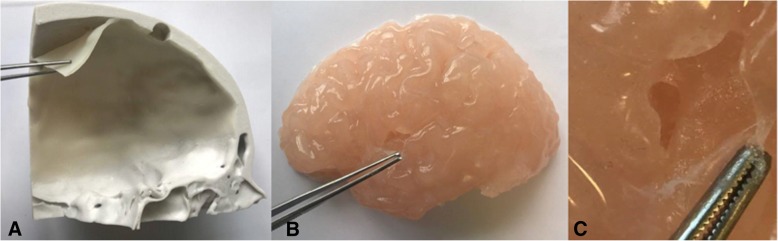
**a** Dura mater model pulled off the calvaria roof demonstrating the thickness and color consistency of the model achieved through silicone application. **b** Brain model (left hemisphere) with pia mater attached and dissected at the Sylvian fissure. **c** Amplified view demonstrating the thin transparent silicone layer consistent with the characteristic of real tissue

Two successive layers of Smooth-On Dragon Skin 10 combined with Silc Pig® pigment additives (PMS 488C, PMS107C and PMS White) was coated onto the deep surface of the replaceable bone component of the skull model until an even distribution of silicone was achieved. Once cured, the silicone was peeled off the skull and the thickness measured using the sliding calipers. The silicone was re-adhered to the skull through the application of Smooth-On Sil-Poxy Silicone adhesive (Fig. 5a).

#### Pia mater

One layer of Smooth-On Dragon Skin 10 combined with Silc Pig® White (PMS White) was brush coated onto the surface of the brain model (left hemisphere) (Fig. 5b-c).

### Skull model

**Fig. 6:**
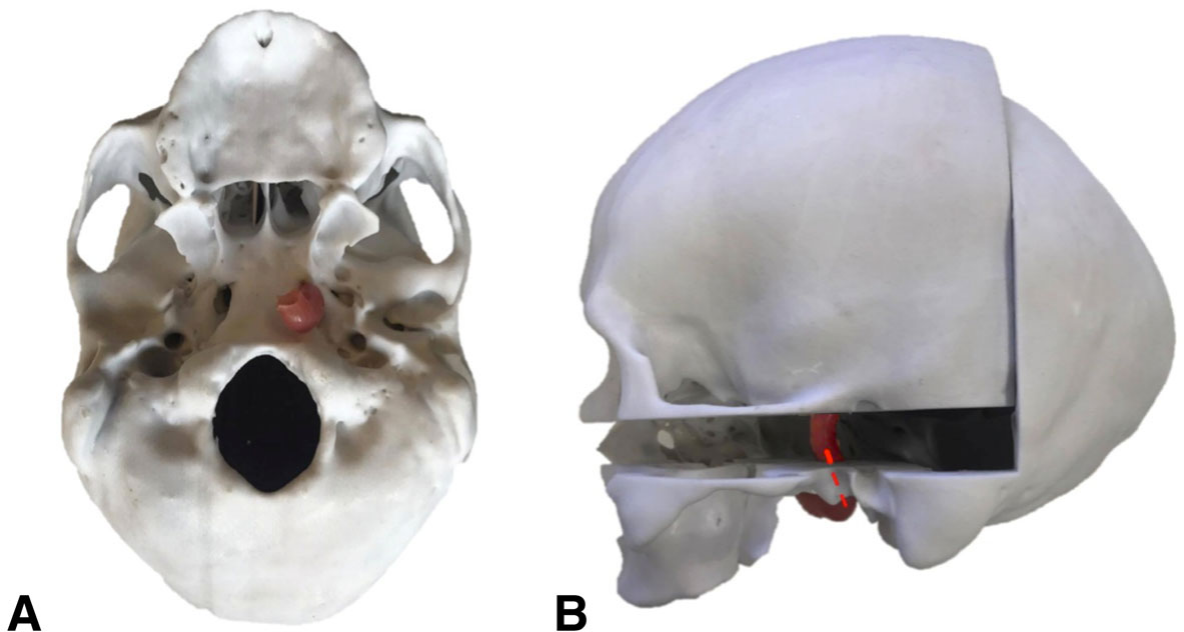
**a** Inferior view and **b** lateral view of the skull model with the vasculature model introduced through the carotid canal across the two skull components (dashed red line)

The simulator skull was produced using two pre-existing computational models derived from CT scans of a cadaveric adult cranium obtained from the CHAE, Department of ADB, Monash University. The two datasets contained anatomical omissions that were rectified through merging the two models using Geomagic Control. The two models were aligned using Manual Registration then trimmed along the same plane superior to the zygomatic process of the frontal bone using Trim With Plane. The subsequent superior and inferior regions were merged using Combine and connected using Fill Single (Flat) tools. The Defeature tool was used to smooth the joined sites. A cube developed in Rhinoceros was used to partition the cranium. The cube was positioned in order for the replaceable component to include all critical anatomy common to pterional and orbitozygomatic craniotomies. The Boolean (Subtract 1/ Intersect) tool was used to produce the cranium and replaceable bone flap component. The two-component skull model was printed on the ZPrinter 650 in a gypsum powder composite material previously reported as similar to that of bone (Fig. 6) [15].

### Skin model

**Fig. 7:**
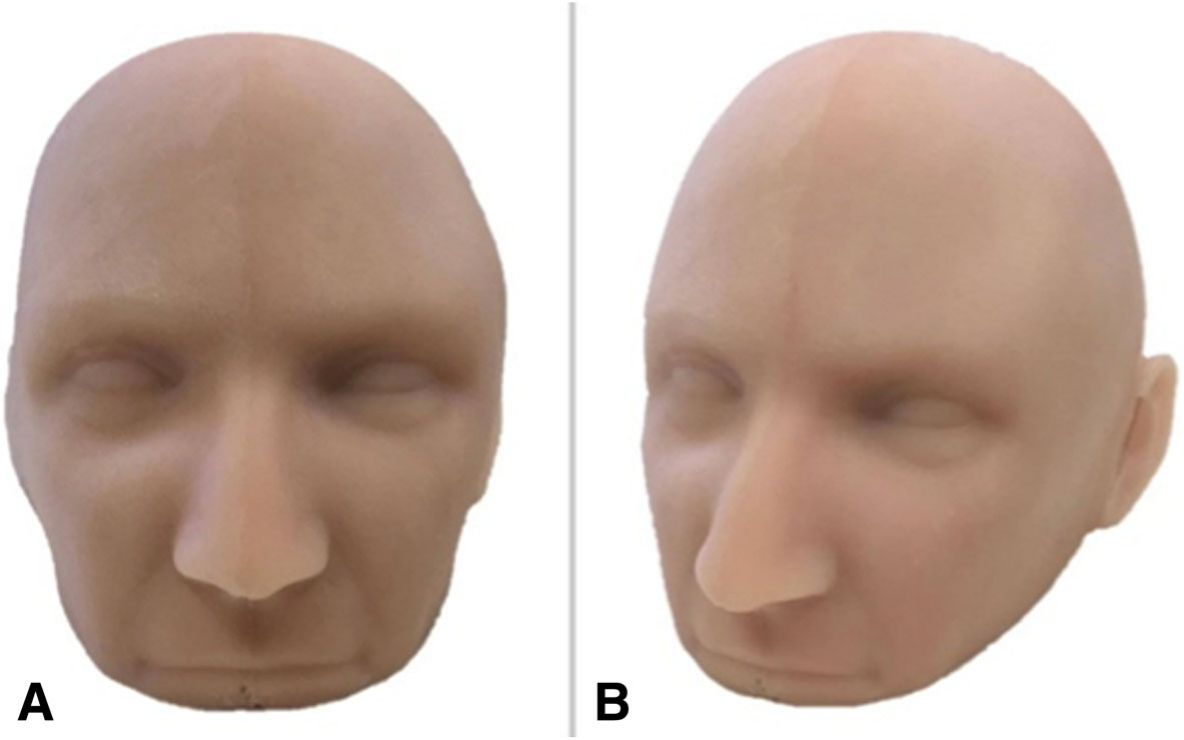
**a** Frontal view of the silicone skin model and **b** angled view permitting visualization of the left ear that serves as a guide for scalp incision during surgical intervention

A full size human head model was downloaded from a free access site (Thingiverse; www.thingiverse.com) as a .STL file. The model incorporated facial anatomy that served as surgical landmarks and improved the fidelity of the simulator. The .STL file was scaled down to correspond with the size of the skull model. A four-part mould was developed using Invert Planes/Flip Normals, Trim With Plane (Intersect Plane) and Combine tools. The mould was printed on the ZPrinter 650, assembled, sealed and cast in Smooth-On Eco-flex 10® with Silc Pig® pigment (PMS 488C) and Surface Tension Diffuser to facilitate demoulding (Fig. 7).

### Accuracy validation

Linear and volumetric measurements of specific physical modular components were repeated, averaged and compared to the computational 3D meshes generated from the medical imaging data. Mean measurements were recorded at defined points of the 3D printed model, cast model and 3D rendered mesh. Measurements included that of nine anthropometric landmarks of the facial skeleton and external surface of the cranial base of the skull model as defined by Aiello and Dean (1990) [17]. Five linear measurements at anatomical sites of the vasculature were measured across the wax and silicone models. All measurements were taken using the sliding calipers. Volumetric measurement of the brain models was determined from the moulds of the left and right cerebral hemisphere. The corresponding measurements were repeated on the 3D meshes using the Geomagic Control Analysis tools. The average of the consecutive measurements was recorded for further calculations.

Calculation of the concordance correlation coefficient was used to measure bivariate pairs of observation, specifically of the 3D mesh and physical models. The statistical analysis was performed on IBM SPSS Statistics 23. A *p*-value less than 0.05 was considered statistically significant. Additional evaluation involved the calculation of the percentage difference between the mean measurements of each mesh and their physical reproductions. These quantitative results are presented in Tables 3, 4 and 5.

**Table 3:** Mean linear measurements of the computational 3D mesh, wax and silicone vasculature model. The percentage difference of the 3D mesh and physical models is presented for each measurement

	Wax Model			Silicone Model		
	Computational Mesh Diameter (mm)	Physical Model Diameter (mm)	Percentage Difference (%)	Computational Mesh Diameter (mm)	Physical Model Diameter (mm)	Percentage Difference (%)
ACA Lumen	1.82	2.55	0.34	1.82	2.66	0.37
MCA Lumen	2.13	2.66	0.22	2.13	2.24	0.05
ICA Lumen	5.13	6.02	0.16	5.13	6.11	0.17
Aneurysm Dome	13.1	13.4	0.03	–	–	–
Aneurysm Neck	5.96	6.13	0.03	–	–	–
ICA to ACA	86.4	87.1	0.01	–	–	–

**Table 4:** Mean volumetric measurement of the computational 3D mesh and the physical brain models (volume of left and right cerebral hemispheres combined). The percentage difference of the 3D mesh and physical model is presented

	Computational Mesh Volume (ml)	Physical Model Volume (ml)	Percentage Difference (%)
Brain (left and right cerebral hemisphere)	1297.1	1293.2	0.0030

**Table 5:** Mean linear measurements of anthropometric landmarks from the computational 3D mesh and the 3D printed skull model. The percentage difference of the 3D mesh and 3D printed model is presented for each measurement

Anthropometric Parameter	Computational Mesh Length (mm)	3D Printed Model Length (mm)	Percentage Difference (%)
Nasal Aperture	32.6	32.4	0.01
Nasospinale to Prosthion	16.6	17.2	0.04
Alveolare to Opisthion	139	139	0.00
Basion to Opisthion	41.8	41.6	0.01
Occipital Condyle	20.6	20.3	0.01
Mastoid Process	99.9	100	0.00
Styloid Process	80.5	80.3	0.00
Pterygoid Hamulus	37.4	37.7	0.01
Foramen Lacerum	32.4	32.2	0.00

Additional quality assurance measures involved reference to the guidelines for medical 3D printing recommended by the Radiological Society of North America (RSNA) 3D printing Special Interest Group (SIG) [18].

## Results

### Vasculature model

Silicone application of two and three layers resulted in a vessel wall thickness of 0.52 mm and 1.40 mm respectively. Two layers, or 0.52 mm, of silicone ensured minimal variance from the true cerebral vascular wall thickness of 0.5 to 0.7 mm at the MCA (Fig. 2a) [19]. Therefore, a two-layer application process was deemed most appropriate.

### CAD aneurysm

Heterogeneity in vascular wall thickness was achieved in which the aneurysm (single layer of silicone) obtained a thinner vascular wall relative to adjacent vasculature (two layers of silicone). The 3D print and wax secondary achieved a defined aneurysm dome and neck following silicone casting (Fig. 2b-d).

### Patient aneurysm

**Fig. 8:**
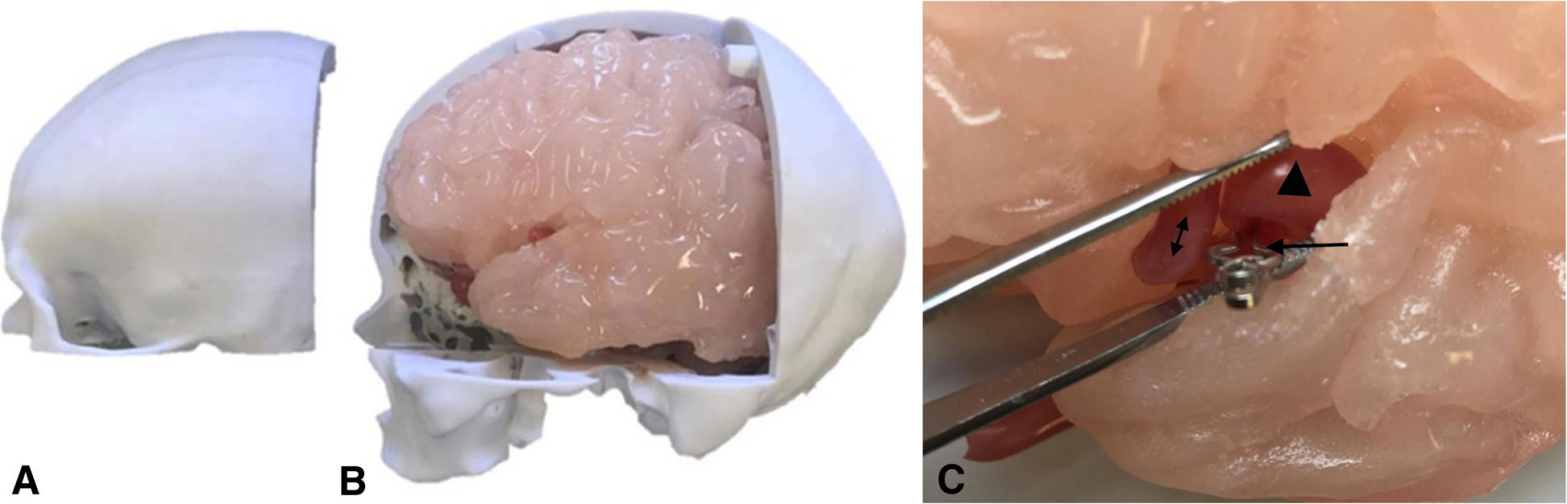
**a** Removal of replaceable bone flap with adhered dura mater model on the interior surface, exposing **b** the left cerebral hemisphere with adhered pia mater model. **c** Retraction of the Sylvian fissure of the brain model and application of a surgical clip (arrow) at the aneurysm neck of the vasculature model. Visualization of the aneurysm dome (triangle) and the neighboring M2 branch (double-sided arrow) of the patient-derived aneurysm model

The left ICA, ACA, MCA and the patient-derived aneurysm at the M2 bifurcating branches were replicated in a hollow, flexible, watertight silicone of an appropriate color (Fig. 8c).

### Circulatory system model

The Waterpik® successfully projected fluid through the vascular model in a pulsatile manner that could be altered by the pressure setting of the apparatus. It permitted visualization of the blood model through the vasculature and slight dilation in the aneurysm dome upon filling of the space with fluid.

### Cranial nerve model

The Polymaker PolyFlex™ produced a replica of the optic chiasm, optic nerve and olfactory tract that was of an appropriate size and color (Fig. 3c).

### Brain model

The medical imaging data lacked the definition required for an effective mould needed to produce a mechanical Sylvian fissure. Editing of the brain mesh achieved a left cerebral hemisphere with a retractable Sylvian fissure and an insular apex in which the M2 aneurysm model could be orientated. Measurements of the Sylvian fissure length and the distance of the insular apex to the surface of the Sylvian fissure, 80.62 mm and 20.30 mm, were close to known average values of 89.40 mm and 19.26 mm respectively [20, 21]. Both hemispheres obtained an even and appropriate color representation when combined with pigment additives (Fig. 4). Both moulds developed a malleable brain model, in particular the left cerebral hemisphere that obtained optimal haptic properties.

### Meninges model

#### Dura mater

The dura mater thickness was measured to be 1.48 mm at the midsagittal edge, 1.43 mm at the coronal edge and 0.99 mm at the inferior edge (Fig. 5a).

#### Pia mater

Brush-coating the silicone onto the brain model produced a replica that adhered to the brain surface and followed the contours of the gyri. The pia mater measured at 0.02 mm in thickness at the Sylvian fissure. The model mimicked the glistening characteristic of pia mater (Fig. 5b-c).

### Skull model

Modelling achieved a replaceable component with relevant anatomy for a craniotomy including portions of the lesser and greater wing of sphenoid, frontal, temporal and parietal bone (Fig. 6). The design facilitated the interchange of interior models. The carotid canal allowed for the introduction of the silicone vascular model through the skull (Fig. 6b). The introduction of two pins allowed for the two components to lock into the correct position.

### Skin model

Silicone and pigment additives produced a cast that retained the facial features of the head mould (Fig. 7).

### Assembly and surgical clipping simulation

The final configuration of the cranial simulator consisted of interchangeable vascular models, optic chiasm, optic nerves, olfactory tract, brain, meninges, skull and skin.

A single surgical clip was applied at the neck of the patient-derived MCA aneurysm model as demonstrated in Fig. 8c. Additionally, the cerebral circulation model was pumped through the vasculature within the brain model and a surgical clip was applied demonstrating the occlusion of the aneurysm from the cerebral circulation.

### Accuracy validation

The measurements of the skull model were within 0.04% deviation of the original computational mesh (Table 5). A statistically significant, almost perfect correlation was determined (ρ_c_> 0.99, *p*-value < 0.01). Volumetric measurement of the physical brain model presented within 0.003% deviation from the original mesh data (Table 4). Aneurysm dimensions and distance of ICA to ACA could not be measured on the silicone model. The enclosure of the aneurysmal lumen within the silicone and the flexible nature of the silicone material prevented the accurate measurement of vessel length. The concordance correlation coefficient for the wax vasculature model indicated an almost perfect correlation (ρ_c_ > 0.99, *p*-value < 0.01) (Table 3). A moderate concordance (ρ_c_ = 0.902, *p*-value > 0.05) was determined for the silicone model, however, this lacked statistical support. Measurements of both the wax and silicone model were within a 0.4% variance from the original computational mesh.

Quantitative measures confirmed the 3D printed models were an accurate replication of input data. The study also adhered to the SIG guidelines. The surface accuracy of the region of interest, specifically the patient-derived aneurysm, was routinely verified using Materialise Mimics. The contours of the final mesh was revealed over the original DICOM images using the software function Contour Visible. The vasculature model including the patient aneurysm consisted of 27,344 triangles, which the study accepted as an appropriate number for the model to adequately represent the imaging data. The resolution of the 3D printers used was superior to that of the medical imaging. All 3D printers manufactured models at a layer thickness of 0.05–0.15 mm, within the SIG recommendation of less than one-third of a millimetre. Post-processing printed models involved the removal of all printing support material and all casting was done in accordance with manufacturer recommendations. The moulding and casting process did not alter the intended morphology of the models with minimal variation in dimensional accuracy. The benefits of casting materials with a reduced shore hardness improved soft tissue representation.

## Discussion

Advances in additive manufacturing technology enable the production of full color, dimensionally accurate, low-cost 3D prints. This technology is being implemented in a range of medical fields [22]. Whilst a variety of substrates are available in 3D printing [23], the haptic properties are currently limited. In addition, limitations in 3D printing technology complicate the manufacture of hollow flexible models and the replication of soft tissues [24]. The present study overcame these limitations through 3D print-driven moulds which allowed us to cast materials that mimicked real tissues. Material selection was based on producing anatomically accurate items with optimal haptic properties.

The unique method of developing a vascular wall through applying various layers of silicone to the wax model was a valuable way of altering vessel wall thickness. The method of brush-coating sequential layers of silicone allowed the representation of aneurysms with a thinner vascular wall than that of the remaining vascular tissue. However, as previously published, hollow silicone models proved difficult to produce [14]. Studies have suggested a rotisserie method of manufacture in which the model is rotated while the silicone cures to ensure an even distribution of material [25, 26].

The final simulator integrated six anatomical models relevant to the treatment of cerebral aneurysms of the Circle of Willis in the left cerebral hemisphere. The collective representation of these anatomical models allowed the simulation of the surgical steps during cerebral aneurysm clipping which included: skin incision and raising the scalp flap; drilling of the burr holes; fashioning and the removal of the bone flap; drilling of lesser wing of the sphenoid bone; durotomy; dissection of the arachnoid plane; opening of the Sylvian fissure; retraction of the left frontal lobe; visualization of the olfactory tract, left optic nerve and optic chiasm; location of the ICA, M1 and aneurysmal neck; and application of surgical clip. The inclusion of these models provided a more complete representation of the entire surgical procedure, broadening the surgical and technical steps that can be simulated. Optimal head positioning in a 3-pin headrest could also be included. As these models were constructed based on patient medical imaging or cadaver-derived computational 3D models they provided a realistic representation of surgically relevant anatomically accurate features. Variability in surgical techniques including the size of the bone flap removed and scalp incision is dependent on surgeon experience and preference. The simulator was designed to cater for this variability with the development of replaceable skull component that expanded beyond the range of standard surgical incisions. The skin also modeled the ears to accommodate for the common performance of a skin incision beginning in front of the ear. The cerebral circulation was demonstrated through the vasculature within the brain model alone to avoid the introduction of input and output tubing extremities that transverse through the head phantom. Future designs are aimed at incorporating the circulation through the entire head.

Critically, our decision to follow a modular design rather than printing via multi-material printing of a single set of nestled files allows for the rapid replacement of worn or damaged components and the potential for models to be interchanged to represent pathological variants. In addition, multiple hollow aneurysm models were developed demonstrating the ability for the single simulator to model various clinical aneurysm cases.

The overall material cost of manufacture was calculated as approximately US $1,280, with a total manufacturing time of 40 h. The majority of the expenses were attributed to 3D printing the cranial base of the skull model and the moulds of the brain model. However, these components are reusable and therefore have a one-time cost of manufacture. Models that are damaged by surgery during simulation cost less than US $22 to replace. Major advantages of the simulator include design adaptability and interchangeable components reducing the cost and time of manufacture.

Quantitative evaluation demonstrated relatively minor variation from the original computational mesh and the physical model counterparts. The vasculature models obtained the greatest percentage difference in comparison to the brain and skull assessments. This increased variation may be accounted for by caliper measurements obtaining greater error at smaller sizes [27]. Likewise, the calculated concordance correlation coefficients indicated moderate to almost perfect correlations. The limited data available on the mechanical properties of human tissues on an equivalent scale complicate the comparison of our models to their respective tissue type. Currently, there are no models that precisely replicate the biomechanical qualities of human tissue [24]. The SIG quality assurance measures were maintained in order to uphold mechanical and geometric integrity. The gap in the literature highlights the demand for such comparative research particularly as the field of medical 3D printing expands.

Previous studies have tested 3D printed models through the simulation of procedures by neurosurgeons and trainees [11, 15, 28, 29]. These studies are often qualitatively validated by a Likert questionnaire provided to participants following simulation [15, 30]. Further study, involving a more complex evaluation method would provide more rigorous validation about the fidelity, educational value and clinical feasibility of the presented simulator.

The vascular model presented here provides a means of simulating endovascular interventions as the vascular lumen represents accurate parameters as it was derived from patient medical imaging, as well as, the inclusion of part of the cerebral circulation. It is feasible for endovascular instruments to be introduced to the vasculature model, however, we have not assessed it for interventional radiology. Further assessment of our simulator with neurosurgical trainees will be essential to assess the endovascular applications of the simulator. Our simulator could easily be adapted for aneurysms in other sites such as the anterior communicating artery.

Hurdles still exist before the bespoke ‘bedside’ application of additive manufacturing is broadly applied in pre-surgical training particularly in cases where multiple tissue-types are represented. The proposed manufacturing method required extensive time to extract radiographic data, create 3D digital models, 3D print and finally assemble the simulator. Such a method would only be applicable for surgical cases that allow for appropriate preparation time.

The incorporation of 3D printing laboratories is increasingly occurring in clinical medicine and surgery in a variety of ways. The use of 3D printing for pre-operative planning is being more broadly adopted in hospital-based care. The present study demonstrates a further area in which 3D printing may impact patient care by allowing the production of more accurate simulation devices. The design flexibility and modular design of a standardized simulator overcomes many of the manufacturing limitations.

## Conclusions

The applications of 3D printing in medicine are enhanced when integrated with real patient medical imaging data. In this study 3D printing was complemented with the casting of synthetic materials to achieve bio-mimicry properties. The design adaptability we have shown is a major advantage as it allows the development of modular customized simulators to meet a range of teaching and training scenarios.

## Data Availability

The datasets used and/or analyzed during the current study are available from the corresponding author on reasonable request.
